# Histone Deacetylase 1 Plays an Acetylation-Independent Role in Influenza A Virus Replication

**DOI:** 10.3389/fimmu.2017.01757

**Published:** 2017-12-12

**Authors:** Lin Chen, Chengmin Wang, Jing Luo, Wen Su, Meng Li, Na Zhao, Wenting Lyu, Hamidreza Attaran, Yapeng He, Hua Ding, Hongxuan He

**Affiliations:** ^1^National Research Center for Wildlife Born Diseases, Institute of Zoology, Chinese Academy of Sciences, Beijing, China; ^2^University of the Chinese Academy of Sciences, Beijing, China; ^3^Department of Infectious Diseases, Hangzhou Center for Disease Control and Prevention, Hangzhou, China

**Keywords:** influenza A virus, nucleoprotein, deacetylation, histone deacetylase 1, type I interferon

## Abstract

Influenza A viruses (IAVs) take advantage of the host acetylation system for their own benefit. Whether the nucleoprotein (NP) of IAVs undergoes acetylation and the interaction between the NP and the class I histone deacetylases (HDACs) were largely unknown. Here, we showed that the NP protein of IAV interacted with HDAC1, which downregulated the acetylation level of NP. Using mass spectrometry, we identified lysine 103 as an acetylation site of the NP. Compared with wild-type protein, two K103 NP mutants, K103A and K103R, enhanced replication efficiency of the recombinant viruses *in vitro*. We further demonstrated that HDAC1 facilitated viral replication *via* two paths: promoting the nuclear retention of NP and inhibiting TBK1-IRF3 pathway. Our results lead to a new mechanism for regulating NP acetylation, indicating that HDAC1 may be a possible target for antiviral drugs.

## Introduction

Influenza A viruses (IAVs) are common pathogens that pose a severe threat to public health (1–4). IAVs consist of eight negative-sense, single-stranded genomic RNA segments, encoding up to 17 proteins (5–8), among which polymerase basic protein 1 (PB1), PB2, polymerase acidic protein (PA), and nucleoprotein (NP) form viral ribonucleoprotein (vRNP) complex, which is responsible for both transcription and replication of viral genome.

Among the four genes within vRNP, NP encodes 498 amino acids and is relatively more conserved. In forming the RNP, NP also interacts with PB1 and PB2 subunits in the vRNP polymerase (9, 10). Besides, NP is suggested to be a vital adaptor for virus and host–cell interaction (11, 12). It has been reported that NP interacted with importin α, F-actin, and CRM1 (9). Previous studies reported that NP undergoes four posttranslational modifications: phosphorylation by cellular kinases (13, 14) and modification by ubiquitination, sumoylation, and ISGylation (15–17). NP is a monoubiquitinated protein and K184 is the ubiquitination site of NP, which is crucial for virus RNA replication (15). Acetylation is an important posttranslational modification of proteins (18); however, no clear evidence has been provided to show that NP is acetylated. The actual modification sites of NP and the role, if any, of NP acetylation in virus infection remain unknown.

Acetylation is a reversible posttranslational modification that has been studied extensively in histones to gain understanding of chromatin structure and gene transcription (19, 20). The transfer of acetyl groups to and from histones is controlled by histone acetyltransferases and histone deacetylases (HDACs), which have opposing activities (21). HDACs are a family of enzymes that catalyze the deacetylation of acetylated protein, consequently influencing diverse cellular processes, such as chromatin remodeling, RNA splicing, gene expression, cell cycle, protein stability, and transport (22–24). So far, a total of 18 HDACs have been identified and grouped into four classes, Class I includes HDAC1, 2, 3, and 8, and class II contains HDAC4, 5, 6, 7, 9, and 10. Class III comprises the Sir2-like deacetylases Sirt1 to 7. HDAC11 is in a unique Class IV (21). HDACs play important roles in virus replication and pathogenesis during infection with a lot of human pathogens, including hepatitis B and C viruses, herpesviruses, HPV and HIV-1 (25–27). Further analyses of HDAC substrates in virus infection will facilitate define acetylation-dependent mechanisms involved in host response to viruses, providing new targets for the development of antiviral therapeutics. It has been reported that HDAC1 and HDAC6 negatively regulated influenza virus infection (28, 29). In their reports, depletion of HDAC1 in A549 cells increased infectivity more than twofold compared with controls. Nagesh et al. have shown that IAVs downregulate HDAC1 expression at both the mRNA and protein level (30). However, the interplay between the viral proteins and HDAC1 and the phosphorylation of interferon regulatory factor 3 (IRF3) and production of IFNα in HDAC1-depleted cells are unknown.

In this study, we found that NP interacted with HDAC1, and HDAC1 regulated NP acetylation *via* the acetylation site at the lysine 103 (K103) of NP. Knockdown of HDAC1 depressed the infection through activation TBK1-IRF3 pathway. These findings may provide an alternative new innate immunological mechanism against influenza virus.

## Materials and Methods

### Cell Lines and Viruses

Human embryonic kidney 293T, A549, and MDCK cells were obtained from ATCC. Cells were grown in Dulbecco’s modified Eagle’s medium (DMEM) supplemented with 10% FBS and 1% penicillin/streptomycin in an incubator at 37°C with 5% CO_2_. The influenza viruses used in this study, A/environment/Qinghai/1/2008(H5N1), A/Beijing/05/2009(H1N1), and A/Chicken/Hangzhou/174/2013(H7N9), were isolated and stored in Institute of Zoology, Chinese Academy of Sciences. The Viruses were propagated in 10-day-old specific pathogen-free embryonated chicken eggs. All experiments with IAVs were carried out in biosafety level 3 containment laboratories approved by the Chinese Academy of Science.

### Plasmids

The full-length NP, PB1, PB2, PA, HA, NA, NS, M [A/environment/Qinghai/1/2008(H5N1)] was cloned into the pHW2000 vector, respectively. The full-length coding sequence of NP was cloned into the pcDNA3.1-Myc-His and pET-28a vectors. Lysine (K) to alanine (A) or arginine (R) mutation in the NP gene was introduced by using the QuikChange site-directed mutagenesis kit (Stratagene) following the manufacturer’s protocol. The full-length coding region sequences of human HDAC1, HDAC2, HDAC3, and HDAC8 were amplified from the HEK293T cDNA and then inserted into the pcDNA3.0-HA and pGEX-4T-1 vectors. All primers used in this study are shown in Table S1 in Supplementary Material. All plasmids were confirmed by Sanger sequencing at BGI (Shenzhen, China).

### Purification of Recombinant Proteins

Glutathione S-transferase (GST) fusion protein GST-HDAC1 and 6His fusion protein 6His-NP were expressed in *E. coli* strain BL21 host cells induced by 0.5 mM IPTG for 4 h at 28°C. After cell lysis and centrifugation, the GST-HDAC1 and 6His-NP proteins were purified with glutathione-sepharose 4B and nickel-nitrilotriacetic acid (Ni-NTA) column (QIAGEN, Gaithersburg, MD, USA) as reported previously (31, 32), respectively. The purified proteins were concentrated and dialyzed against PBS. The recombinant proteins were kept at −80°C until use.

To purify the acetylated NP protein, 293T cells were transfected with NP-6His and then treated with 1 µM trichostatin A (TSA) for 24 h, cell extracts were purified by Ni-NTA column as described previously (33). In brief, cell extracts were incubated with Ni-NTA beads for 4 h at 4°C. Beads were then washed with buffer A (20 mM Tris-HCl (pH8.0), 0.5 M Nacl, 10 mM Imidazole), and the bound proteins were eluted with buffer B (20 mM Tris-HCl (pH8.0), 0.5 M Nacl, 300 mM Imidazole). The eluent NP proteins were then concentrated and dialyzed against PBS.

### *In Vitro* Deacetylation Assay

The deacetylation assay was performed as described previously (34). Briefly, the acetylated NP was incubated with GST-HDAC1 in HDAC assay buffer (25 µM Tris-HCl (PH8.0), 137 mM NaCl, 2.7 mM KCl, 1 mM MgCl_2_, and 60 µM NAD^+^) in a final volume of 50 µl for 60 min at 30°C. The proteins were resolved by 12% SDS-PAGE and analyzed by Western blot.

### Antibodies

The antibodies used for immunoblotting include: mouse monoclonal antibodies (mAbs) c-Myc antibody (sc-56634) and β-actin (C4) (sc-47778) (both purchased from Santa Cruz Biotechnology, Dallas, TX, USA); rabbit polyclonal antibodies against NP (GTX125989) was purchased from GeneTex (Taiwan); anti-TBK1 (ab40676), and anti-TBK1 (phosphor S172) (ab109272) antibodies were obtained from Abcam (Cambridge, MA, USA). Rabbit polyclonal antibody against acetylated-lysine (9441), mouse mAb against HDAC1 (10E2) (5356), p-IRF3 (4947), IRF3 (4302), p-p44/42 MAPK (Erk1/2) (Thr202/Tyr204) (4370), p44/42 MAPK (Erk1/2) (9102), and rabbit mAb HA-Tag (c29F4) (28) were from Cell Signaling Technology (Beverly, MA, USA).

### Recombinant Viruses

Recombinant viruses were generated by plasmid-based reverse genetics as previously described (35, 36). In brief, The pHW2000-NP plasmid (as wild-type) or NP gene with mutation K103A and K103R was co-transfected with the other seven plasmids pHW2000-PB1, -PB2, -PA, -HA, -NA, -NS, and -M genes from A/environment/Qinghai/1/2008(H5N1) virus into MDCK cells and 293T cells. 24 h posttransfection, the medium was replaced with DMEM plus 1 µg/ml tosylsulfonyl phenylalanyl chloromethyl ketone (TPCK)-treated trypsin. The cells were further cultured for 72 h, and the supernatant containing the recombinant viruses was harvested. The K103A and K103R mutations of NP in the generated viruses were confirmed by sequencing.

### Plaque Assay

MDCK cell monolayers (5 × 10^6^ cells at 100% confluence in a 6-well plate) were infected with different dilutions of virus for 1 h at 37°C, with shaking every 15 min. The virus inoculums were then removed, whereas the infected cells were washed by PBS, and covered with agar overlay medium (DMEM supplemented with 1 µg/ml TPCK-treated trypsin and 0.6% low-melting point agarose) and then incubated at 37°C for 2–3 dpi. Visible plaques were counted and virus titers were determined. All data were expressed as means of three independent experiments.

### Growth Curves

To characterize, the single growth curves of rWT, rK103A, and rK103R viruses on A549 cells, we first titrated the viruses on MDCK cells and then used them to infect A549 cells at an MOI of 1. After 1 h incubation at 37°C in 5% CO_2_, the medium were replaced with DMEM with 2% FBS, and the cell-free supernatants were harvested at 3, 6, 9, and 12 h postinfection. Virus amounts in the supernatant were determined using MDCK cells. For the multiple growth curves, the A549 cells were infected with rWT, rK103A, or K103R virus at an MOI of 0.1, and the procedures were the same as those for the single growth curve experiments.

### Luciferase Reporter Assay

To determine the polymerase activities of the ribonucleoprotein complexes, 293T cells were co-transfected with 1 µg of each pHW2000 plasmid encoding the polymerase subunits PB2, PB1, PA, and NP (WT or mutants), together with 1 µg pPOL1-Luc and 50 ng pRL-TK. Renilla luciferase was used as an internal control. The transfected cells were incubated at 37°C and harvested at 36 h posttransfection, and luciferase levels were measured by using the Dual Luciferase Assay System (Promega, Madison, WI, USA) according to the manufacturer’s protocol.

### RNA Isolation, Reverse Transcription (RT), and qPCR

Total intracellular RNA was extracted by using TRIzol (Invitrogen, Carlsbad, CA, USA). The RT reaction was carried out by using the GoScript Reverse Transcription System (Promega, Madison, WI, USA). For synthesizing the cDNA, we used the influenza A-specific primer uni-12 (5′-AGCAAAAGCAGG-3′) and random primer.

Each cDNA sample was analyzed in triplicate on an ABI 7500 Real-Time Detection System (Applied Biosystems) using SYBR Green (CWBio, Beijng, China) according to the manufacturer’s protocol. Endogenous housekeeping genes (β-actin or GAPDH) were used as internal standards. Cycle conditions were as follows: 95°C for 2 min followed by 40 cycles of 95°C for 15 s and 60°C for 1 min.

### Immunoprecipitation and Western Blot Analysis

Cells were lysed in RIPA buffer (50 mM Tris-HCl pH 7.6, 150 mM NaCl, 0.1% SDS, 1% Triton-100) supplemented with protease inhibitor (Roche, Basel, Swiss) and 300 nM TSA. Protein concentrations of the extracts were determined by bicinchoninic acid assay. NP was purified with protein A Mag Sepharose (GE Healthcare, Little Chalfont, UK) prebound to a mouse Myc-tagged mAb for 10 h at 4°C. The immunocomplexes and the whole cell lysate were resolved by SDS-PAGE. Proteins were transferred to nitrocellulose membrane and blocked with 5% nonfat milk in TBS with 0.1% Tween 20 (TBST). The membrane was probed with primary antibody followed by secondary antibody at room temperature for 2–4 h. The membrane was stripped with stripping buffer before being probed again with another antibody.

### Generation of HDAC1 Knockdown Cells

The production of lentivirus expressing HDAC1–shRNA\vector was purchased from Sigma-Aldrich (TRCN0000195103 and TRCN0000004818). The clone of short hairpin HDAC1 (shHDAC1) used for knockdown of HDAC1 was validated. The lentivirus-negative control was generated from pLKO.1, in which the shRNA sequence was replaced by the following scrambled sequence: 5′-AGACGTGGTTTTTTTTGCTAGCTTGC-3′. A549 cells were infected with shHDAC1 lentivirus for 48 h, and then the medium was replaced with selective medium containing puromycin (2 µg/ml). After the cells were incubated for 14 days, individual clones were obtained, expanded, cryopreserved, and cultured in 6-well plates. The total cell lysate was collected to check the knockdown efficiency of shHDAC1 by Western blot.

### HDAC1 Small Interfere RNAs (siRNAs) and Transfection

The siRNA duplexes against HDAC1 and the negative control siRNA (siCONT) were purchased from Invitrogen (Shanghai, China). The sequences of the two HDAC1 siRNAs are as follows: CUGUACAUUGACAUUGAUAdTdT (siHD1-1) and CAGCGAUGACUACAUUAAA dTdT (siHD1-2). The siRNAs transfection was performed using Lipofectamine 3000 (Invitrogen) according to the manufacturer’s instruction.

### Statistical Analysis

Student *t*-test (where two groups of data were compared) or two-way ANOVA followed by Bonferroni post-test analysis (where more than two groups of data were compared) was used for statistical comparisons. Data were expressed as means ± SDs. A *P* value of 0.05 or less was considered statistically significant.

## Results

### HDAC1 Interacted with NP *In Vivo* and *In Vitro*

**Figure 1:**
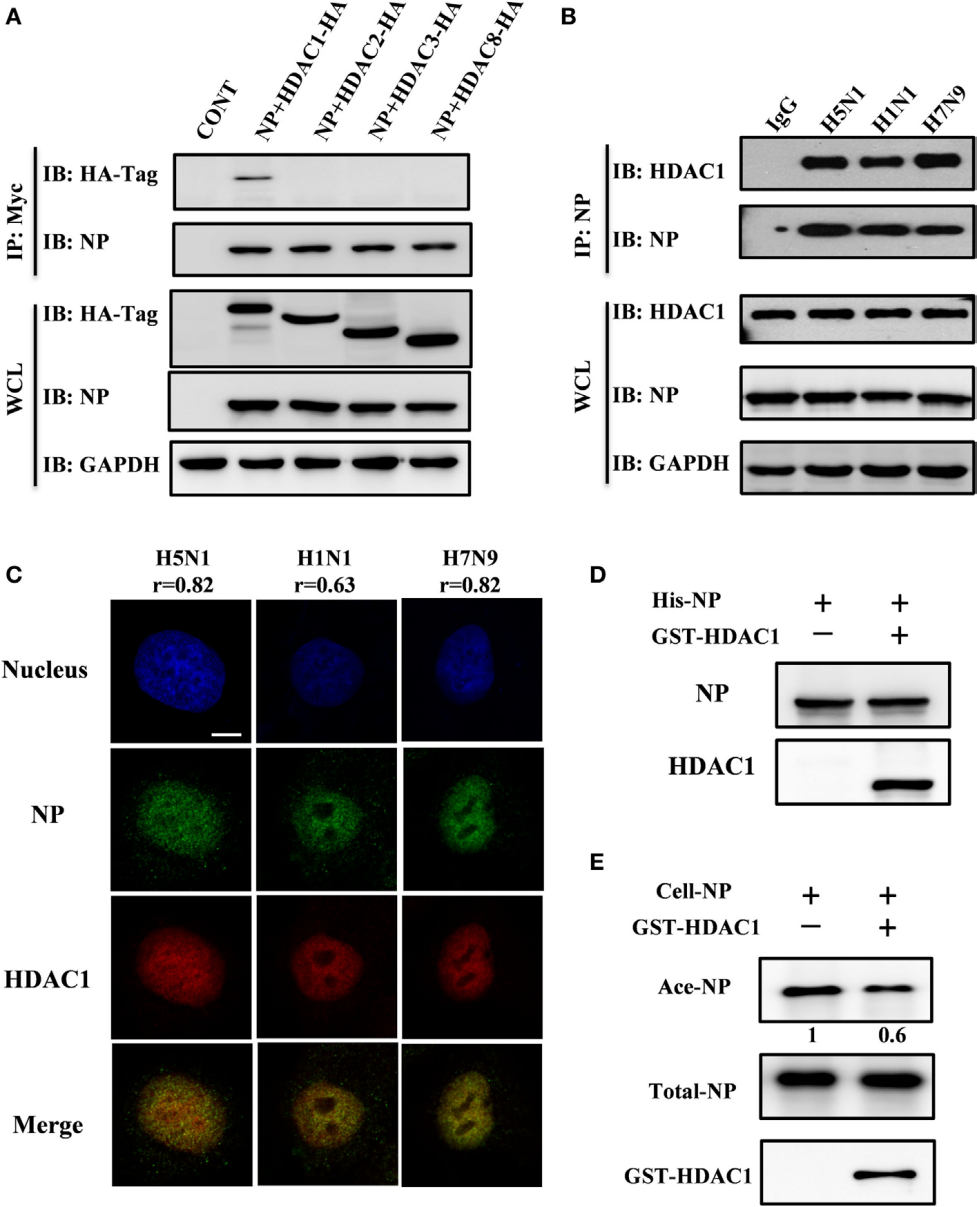
Nucleoprotein (NP) interacted with histone deacetylase 1 (HDAC1) *in vivo* and *in vitro*. **(A)** Myc-NP was co-expressed with HA-tagged HDAC1, HDAC2, HDAC3, or HDAC8 in HEK293T cells, and immunoprecipitates by anti-Myc antibody. The samples were blotted with indicated antibodies. GAPDH was used as a loading control. **(B)** 293T cells were infected with H5N1, H1N1, or H7N9 virus at an MOI of 1. 24 h postinfection, the cell lysates were subjected to immunoprecipitation with control IgG or NP antibodies and subsequently immunoblotted with indicated antibodies. **(C)** A549 cells were infected with H5N1, H1N1, or H7N9 virus at an MOI of 1. 12 h postinfection, cells were fixed and stained by NP antibody (green) and HDAC1 (red). The Pearson’s correlation (r) of NP-HDAC1 co-localization was calculated by Imaris, at least 40 cells in each group were scored. The nucleus was stained with DAPI (blue). Scale bar: 5 µm. Data are representative of three independent experiments. **(D)** 6His-NP (20 µg) immobilized on nickel-nitrilotriacetic acid (Ni-NTA) beads were incubated with/without recombinant HDAC1 (20 µg), the beads were then extensively washed, and the proteins resolved by SDS-PAGE and detected the NP and HDAC1 by Western blot. **(E)** *In vitro* deacetylation assay. 293T-expressed NP-6His proteins (5 µg) were purified and washed with high salt buffer to remove the binding partners, then incubated with glutathione S-transferase (GST)-HDAC1 (1 µg) in HDAC buffer for 60 min at 30°C. The proteins were resolved by SDS-PAGE and detected the Ace-NP, NP, and HDAC1 by Western blot.

Class I HDACs (i.e., HDAC1, HDAC2, HDAC3, and HDAC8) play a critical role in the life cycle of influenza virus (28). To determine which of these HDACs binds to NP, we co-transfected each Hemagglutinin (HA)-tagged HDAC with Myc-tagged NP. At 36 h after transfection, the cell lysates were subjected to immunoprecipitation with Myc-tagged antibody. Immunoblot analysis, using HA-tagged antibody, showed that NP interacted with HDAC1 but not with HDAC2, HDAC3, or HDAC8 (Figure 1A). The viral NP came from H5N1, H1N1, and H7N9 viruses in infected HEK293T cells was also demonstrated to interact with endogenous HDAC1 by the co-immunoprecipitation by anti-NP antibody (Figure 1B). And the binding was validated by the nucleus co-localization of NP and HDAC1 in H5N1, H1N1, and H7N9 viruses infected A549 cells (Figure 1C). The co-localization is quantified using Pearson’s correlation (r) (0.8, 0.6, and 0.8 for H5N1, H1N1, and H7N9, respectively). To better define, the direct interaction between NP and HDAC1, a Ni-NTA pulldown assay was carried out using 6His-NP and GST-HDAC1 expressed in *E. coli* strain BL21. As shown in Figure 1D, NP interacted with the recombinant HDAC1 *in vitro*. Furthermore, HDAC assay *in vitro* showed that the acetylation level of NP (Ace-NP) was decreased about 40% when the acetylated NP was incubated with GST-HDAC1 (Figure 1E). Taken together, our results demonstrated the NP protein interacted with the HDAC1 *in vivo* and *in vitro*, and the cellular NP was deacetylated by recombinant HDAC1 *in vitro*.

### The Conserved K103 of NP Protein Is a Target Site Regulated by HDAC1

**Figure 2:**
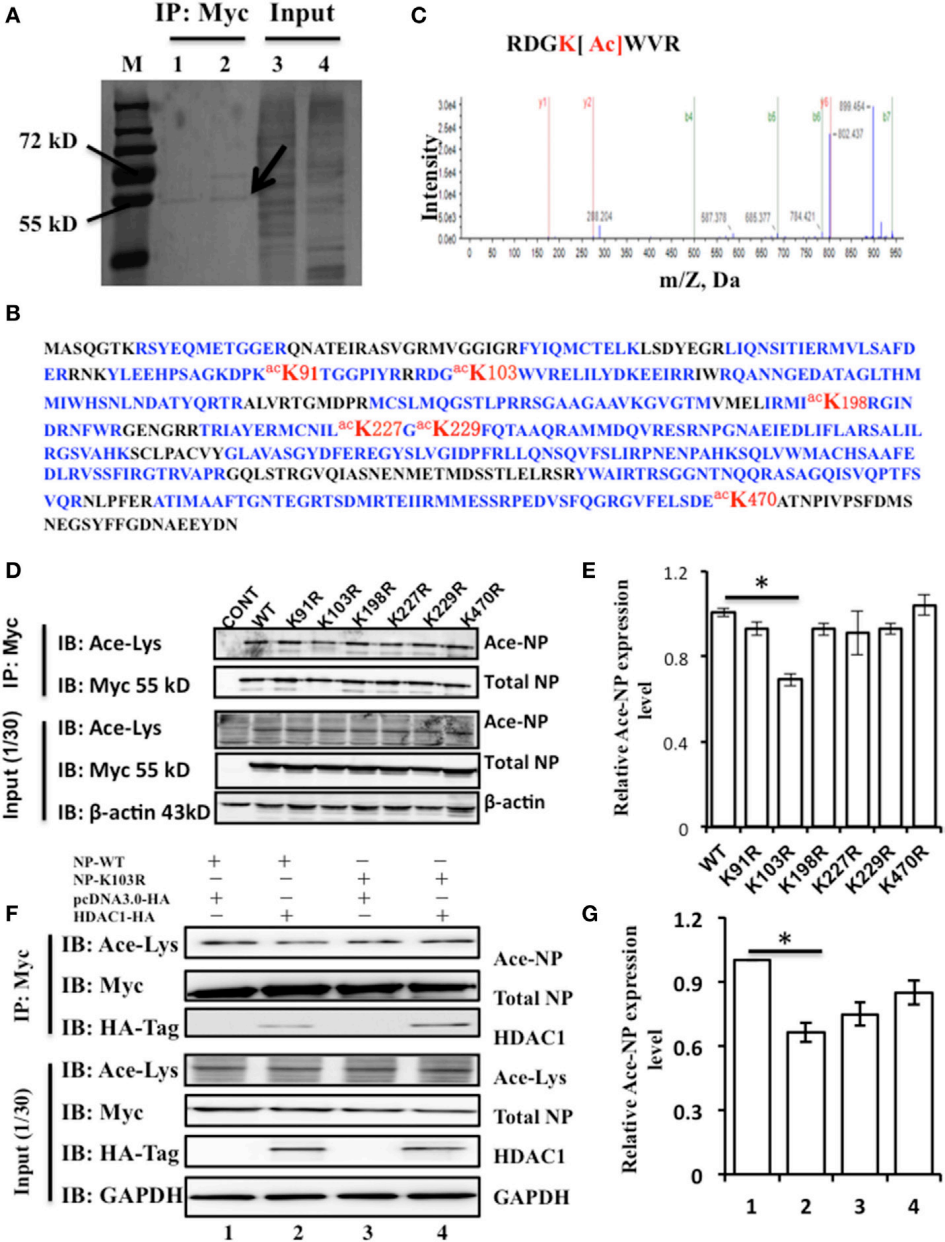
Nucleoprotein (NP) is acetylated on the lysine residue at amino acid position 103. **(A)** HEK293T cells were transfected with the NP with a Myc-tagged at the C-terminal. The Myc-NP (red arrow indicated) band was analyzed by mass spectrometry. **(B)** The protein was identified as the influenza A viruses (IAV) [A/environment/Qinghai/1/2008(H5N1)] NP. Blue indicates peptides detected by mass spectrometry; red indicates the acetylated lysine sites. **(C)** K103 is the primary acetyl amino acid suggested in mass spectrometry. **(D)** The expression of lysine to alanine substitution mutants of NP. HEK293T cells were transfected with NP-Myc WT or mutants. WT and mutant NP were purified by Myc-tag pull-down followed by Western blotting (IB) with the anti-myc antibody (Myc) or anti-acetylated lysine antibody (Ace-Lys). β-actin was used as a loading control. **(E)** The relative expression level of Ace-NP protein in **(D)** was quantified by imageJ. **(F)** Histone deacetylase 1 (HDAC1) deacylated the acylation of NP at K103. HEK293T cells expressing indicated proteins were subjected to immunoprecipitation using anti-Myc antibody, the samples were immunoblotted with the indicated antibodies. β-actin was used as a loading control. **(G)** The relative Ace-NP protein expression level in **(F)** was quantified by using imageJ. Statistical significance was determined by Student’s *t-*test. *P < 0.5 versus WT group. Data are representative of three independent experiments [mean ± SD in **(E,G)**].

Above results showed that NP interacts with HDAC1, but whether NP is an acetylated protein or the actual modification sites of NP were need to explore. To identify the acetylated amino acid residues within the NP, 293T cells were transfected with NP plasmids with a Myc-tagged. Transfected cell proteins, that were either treated or non-treated with TSA (300 ng/ml) for 24 h, were purified using Myc-tagged antibody. TSA was used to suppress the activity of HDACs and expose the potential acetylation sites. The immunoprecipitation complexes were then resolved by SDS-PAGE and stained with Coomassie blue. A visible band with a molecular mass of 55 kD (Figure 2A), was extracted and subjected to liquid chromatography-tandem mass spectrometry, and results confirmed this band as the NP of influenza A/environment/Qinghai/1/2008(H5N1) virus (Figure 2B). Four acetyl-lysine residues, K103, K227, K229, and K470, were detected without TSA treatment, and two additional acetyl lysine residues, K91 and K198, were detected after TSA exposure (Figure S1A in Supplementary Material). Further analysis of the spectra indicated that the peptide containing K103 was acetylated at this position (Figure 2C).

To further determine the functional acetylation sites on NP, we performed site-directed mutagenesis to replace the K residue at one of six sites with arginine (R), which will remove the acetylation if there is any. We transfected HEK293T cells with the NP mutants (K91R, K103R, K198R, K227R, K229R, and K470R) and purified them by using Myc-tagged antibody and detected the Ace-NP using the Ace-lysine antibody. The results showed that K103R reduced the 30% level of NP acetylation compared with that of the WT protein (Figures 2D,E). With reference to the three-dimensional crystal structure of the NP (Protein Data Bank accession No. 2Q06), it showed that K103 was located on the surface of the NP (Figure S1B in Supplementary Material), indicating the K103 residue had a critical role. Together, our data suggest that the K103 is an acetylation site of NP.

To further determine, whether K103 site was an acetylation site that regulated by HDAC1. HEK293T cells were transfected with NP-WT and NP-K103R expression plasmids with or without HDAC1. The results showed that HDAC1 co-expressed with the NP-WT, and the acetylation was downregulated 0.7-fold compared with that of the control group (Figure 2F). However, the acetylation of NP-K103R was not downregulated (Figure 2G). Collectively, these data suggest that the K103 of NP is an acetylation site regulated by HDAC1.

### Mutations of NP K103 Regulated vRNA Replication and Transcription

**Figure 3:**
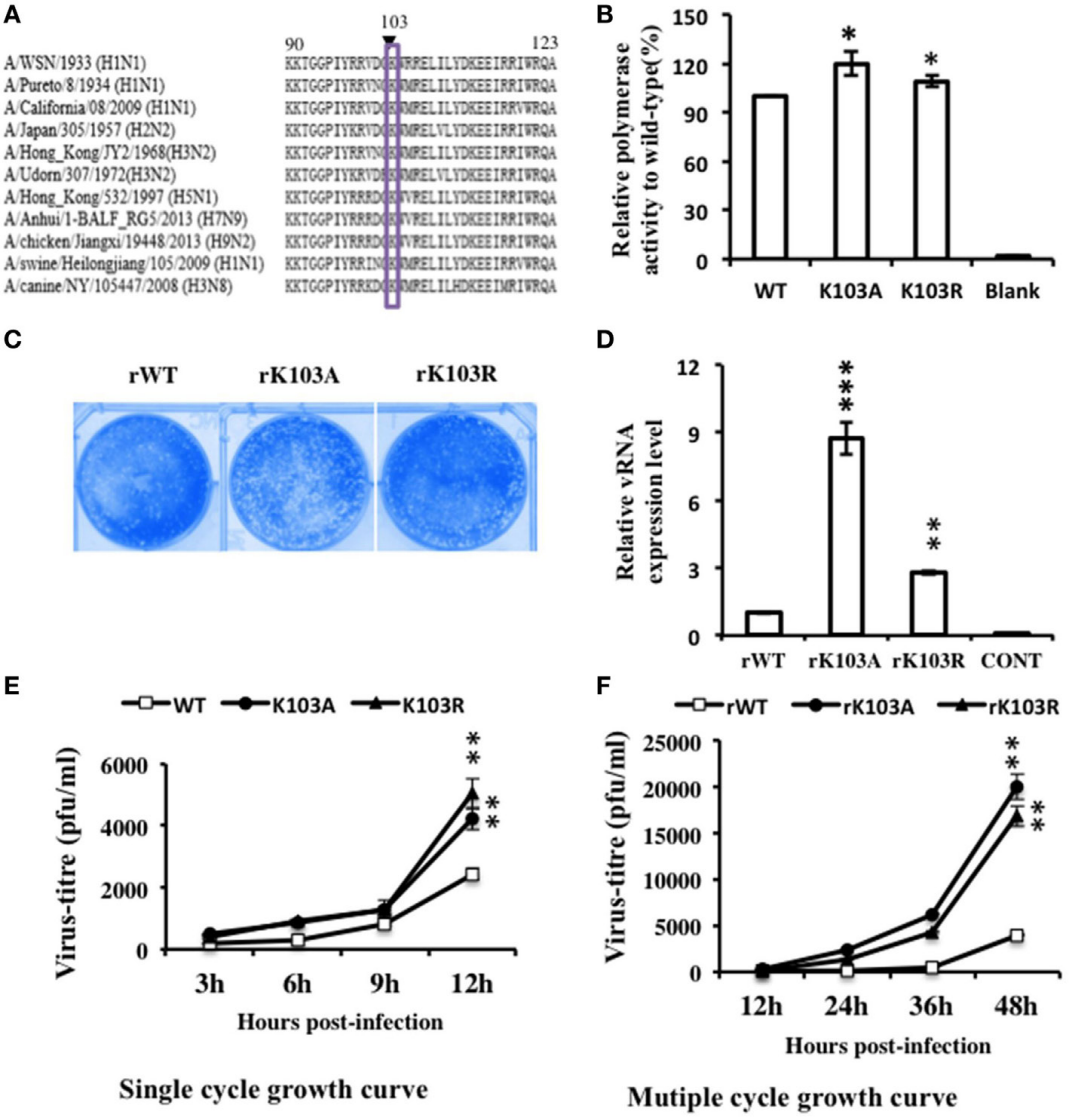
Mutation in K103 promoted the replication of recombinant viruses in *in vitro* assays. **(A)** K103 is highly conserved (residues 90–123) among influenza A viruses (IAV) subtypes. **(B)** Mutation in K103 promoted the replication of IAV RNA genome in mini-replicon reporter system. HEK293T cells were co-transfected with pPolI-Luc, viral protein polymerase basic protein 2 (PB2), PB1, polymerase acidic protein (PA) expression plasmids, with or without nucleoprotein (NP) (WT or mutants). Renilla luciferase was used as an internal control. Data were normalized to the values of WT NP. **(C)** Plaque morphology of NP-rWT, NP-rK103A, and NP-rK103R viruses. **(D)** Mutation in K103 increased the vRNA level. A549 cells were infected with rWT, rK103A, or rK103R virus at an MOI of 1 for 24 h. The vRNA expression level was detected by using primer detected the segment NP. **(E,F)** Mutation in K103 promoted the growth of recombinant viruses. A549 cells were infected with NP-rK103A/R or WT virus at an MOI of 1 **(E)** or 0.1 **(F)**. The supernatant was collected at indicated time after infection, and then, used for plaque assay in MDCK cells. Values are mean ± SD of three separate experiments in **(B,D–F)**. *P < 0.05; **P < 0.01, ***P < 0.001 (analysis of two-way ANOVA followed by Bonferroni post-test).

Sequence alignment showed that K103 is highly conserved among different IAV subtypes (Figure 3A). To assess the roles of K103 in virus life cycle, we introduced K103A/R substitution to mimic the K103 deacetylation state of NP protein. First, we tested the replication function of K103A and K103R by using a mini-replicon system (containing luciferase as a reporter) that assays vRNA replication activity. The results (Figure 3B) showed that the substitution of K103 by A and R increased the RNP activity by 1.4-fold and 1.2-fold, respectively, compared with that of WT. Therefore, these results suggested that K103 mutant increased the RNP activity.

### Recombinant Viruses Containing NP K103 Mutations Increased Virus Replication *In Vitro*

We next examined whether the K103 mutation could affect the growth of the viruses. Recombinant viruses, incorporating the NP-rK103A/R substitution, were produced by reverse genetics techniques (35), and the biological properties of the mutant viruses were examined. The plaque sizes of the NP-rK103A/R mutant viruses were similar to that of the WT virus (Figure 3C). QRT–PCR was performed to detect vRNA expression. 24 h postinfection, vRNA levels of the NP-K103A and NP-K103R mutant viruses were 8-fold and 2.5-fold higher, respectively, than that of the WT virus (Figure 3D). Growth analysis suggested that the virus titer of NP-rK103A/R mutant virus was twofold at 12 h and NP-rK103A fourfold at 48 h postinfection, compared to the rWT virus (Figures 3E,F). Collectively, these data suggest that K103 is a key acetylation site and that the K103A and K103R mutants show increased virus replication levels *in vitro* than that in rWT.

### Depletion of HDAC1 Impaired the Nuclear Retention of NP

**Figure 4:**
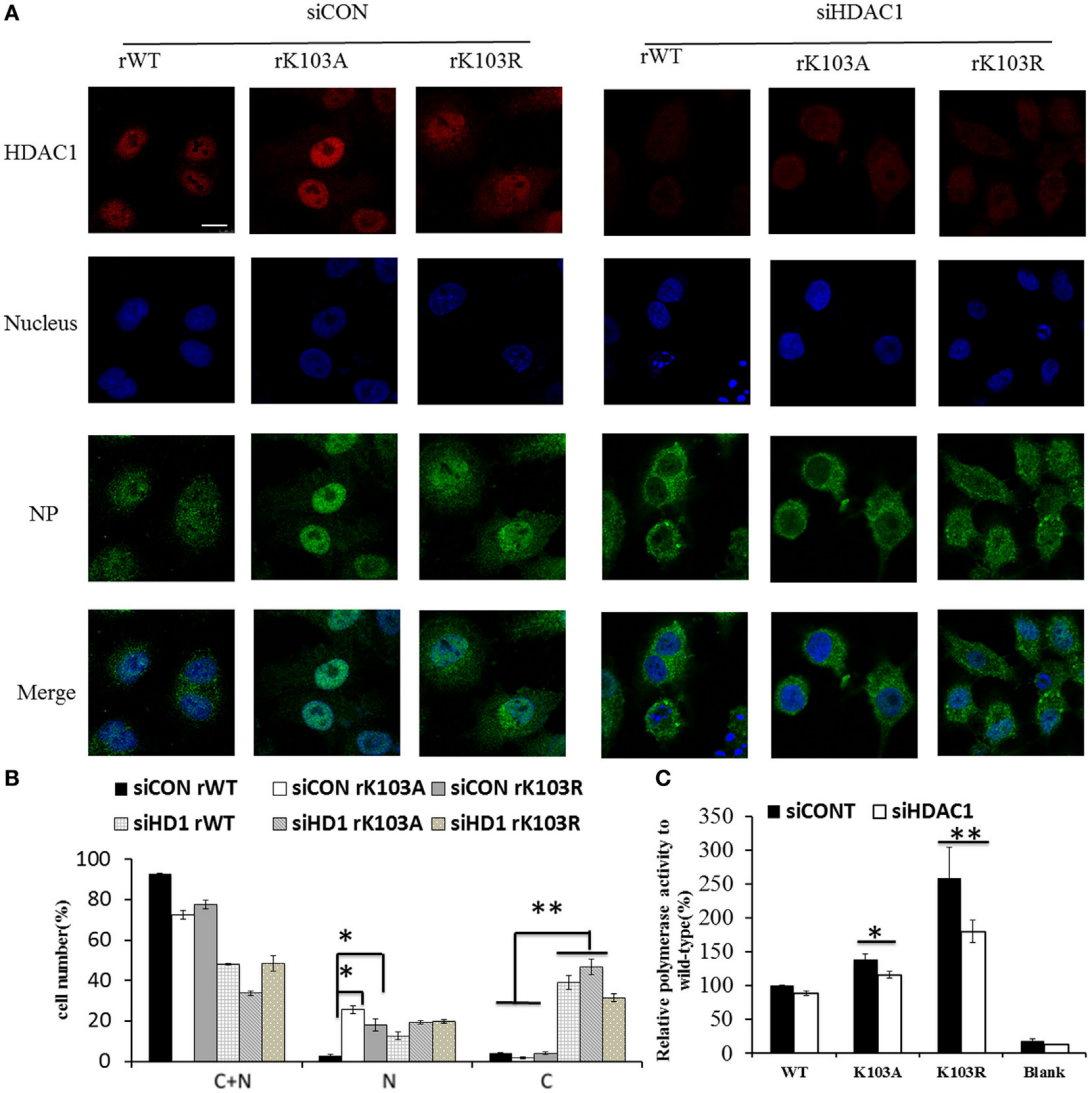
Histone deacetylase 1 (HDAC1) regulated the localization of nucleoprotein (NP) independent the K103 acylation. A549 cells were transfected with siCONT or siHDAC1 for 48 h and then infected with rWT, rK103A, or rK103R viruses at MOI of 1. **(A)** At 24 h postinfection, the subcellular distribution of the NP (green) and HDAC1 (red) was analyzed by IFAs. The nucleus was stained with DAPI (blue). **(B)** More than 400 cells in each group were scored. Nuclear and cytoplasmic (N + C), predominantly nuclear (N), predominantly cytoplasmic (C). *P < 0.05; **P < 0.01 (analysis of two-way ANOVA followed by Bonferroni post-test). Data are representative of three independent experiments [mean ± SD in **(B)**]. Scale bar: 10 µm. **(C)** HEK293T cells were transfected siCONT or siHDAC1 for 48 h and then co-transfected with pPolI–Luc, viral protein polymerase basic protein 2 (PB2), PB1, polymerase acidic protein (PA) expression plasmids, with or without NP (WT or mutants). Renilla luciferase was used as an internal control.

Mutation of K103 may lead to other “gain-of-function” that it may act as a nuclear import/export signal. Therefore, the intracellular distribution of mutant NP expressed from the recombinant viruses was examined by IFAs. Though the NP of rWT, rK103A, and rK103R displayed cytoplasm (C) and nuclear (N) localization, the nuclear fraction of the two mutants was significantly increased (Figures 4A,B). The increased nuclear fraction of rK103A and rK103R may be correlated with the enhancement of viral growth. In addition, when HDAC1 was knockdown in A549 cells, the cytoplasm fraction of NP was significant increased compared with control cells (Figures 4A,B), indicating that HDAC1 facilitated the nuclear retention of NP. Furthermore, depletion of HDAC1 expression resulted in the decrease of the RNP activity (Figure 4C). However, there was no difference between the WT and K103 mutants, suggesting that HDAC1 activity was important for virus replication but this was independent of the deacetylation of the NP K103 residue.

### Depletion of HDAC1 Inhibited Virus Replication through Activation TBK1-IRF3 Signaling Pathway

**Figure 5:**
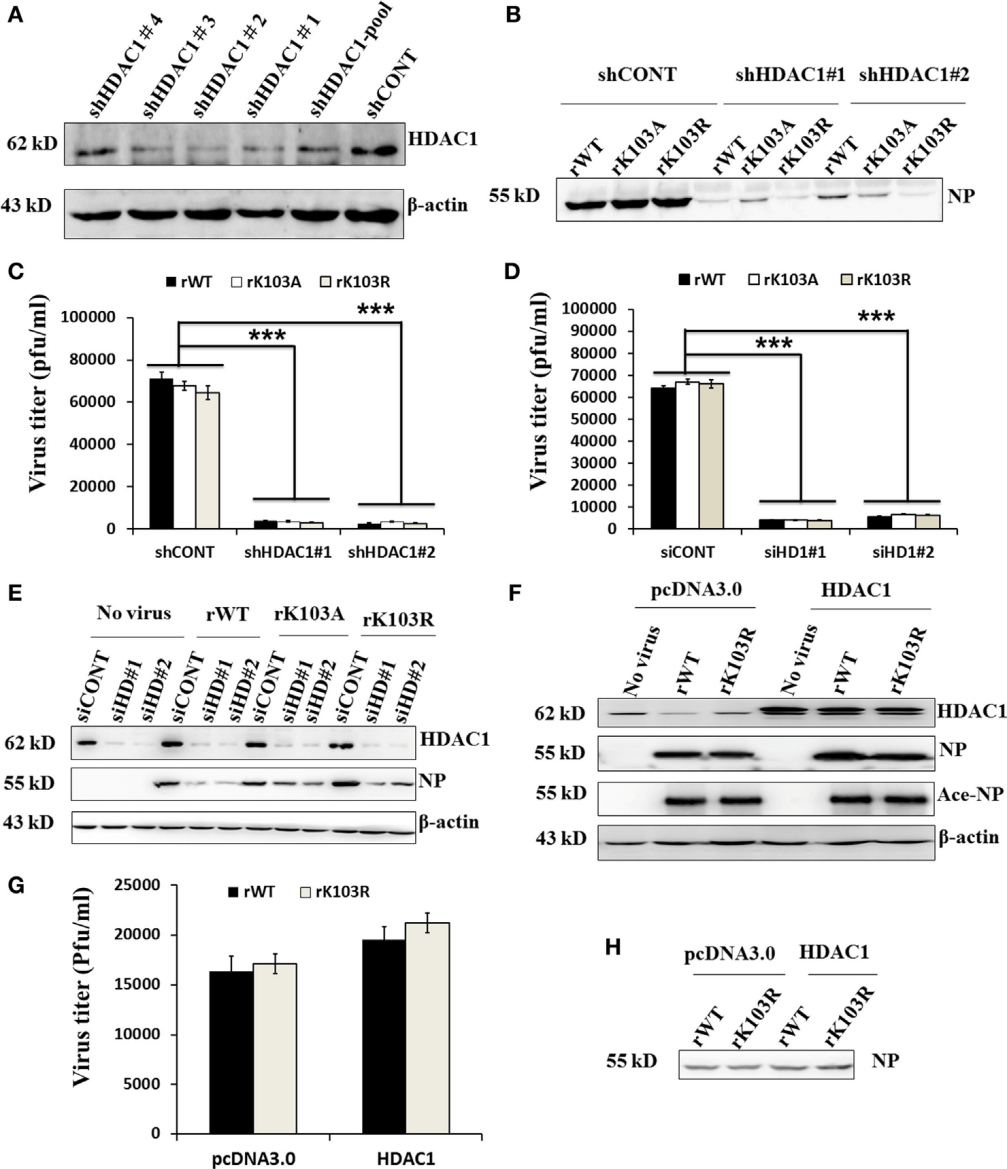
Effects of histone deacetylase 1 (HDAC1) on influenza A viruses (IAV) infection. **(A)** Knockdown of HDAC1 expression was shown by immunoblot analysis, using HDAC1-specific antibody. Four separate short hairpin HDAC1 (shHDAC1) lentivirus clones were used. shCONT was used as the lentivirus infection control. β-actin was used as a loading control. **(B–E)** Depletion of HDAC1 decreased viral nucleoprotein (NP) protein level and inhibited IAV infection. A549 cells with/without shHDAC1 as indicated were infected with NP rWT and mutant virus at an MOI of 1 for 24 h, the virion from the culture medium was collected and subjected to Western blot **(B)** and plaque assay on MDCK cells **(C)**. A549 cells with/without siHDAC1 as indicated were infected with rWT, rK103A, or rK103R virus at an MOI of 1, the virion from the culture medium was collected and subjected to plaque assay on MDCK cells **(D)** and Western blot **(E)**. **(F–H)** Effect of HDAC1 overexpression on IAV infection. A549 cells were transfected with/without pcDNA3.0-HDAC1. 36 h posttransfection, cells were infected with rWT/rK103R virus at an MOI of 1 for 24 h. Total lysate of the infected cells was collected for Western blot **(F)**. The virion from the culture medium was collected and subjected to plaque assay on MDCK cells **(G)** and Western blot **(H)**. *P < 0.05; **P < 0.01, ***P < 0.001 (analysis of two-way ANOVA followed by Bonferroni post-test). Data are representative of three independent experiments [mean ± SD in **(C,D,G)**].

To determine the role of HDAC1 in influenza virus replication, we used shRNA to silence the expression of endogenous HDAC1. Immunoblotting results showed that, among the five shHDAC1 clones, clones 1 and 2, had the best depletion efficiency (Figure 5A). Therefore, these two clones were used for further studies. To verify that HDAC1 is involved in IAV infection, we infected HDAC1 depletion cells with rWT, rK103A, or rK103R influenza virus at an MOI of 1. At 24 h postinfection, the culture medium was collected and divided into two parts; one part was analyzed by Western blot and the other was analyzed by plaque assay to determine virus titers. Depletion of HDAC1 resulted in decreased NP and a lower virus titer (Figures 5B,C). The result was also verified in transient HDAC1 knockdown cells using siRNA (siHD1#1 and siHD1#2) (Figures 5D,E). Intriguingly, overexpression of HDAC1 in A549 cells only slightly increased virus production and virus titer (Figures 5F–H). It is possible that the endogenous HDAC1 was enough for the replication of virus and the effect of extraneous HDAC1 was limited. Taken together, these results suggest that HDAC1 can regulate IAV production.

**Figure 6:**
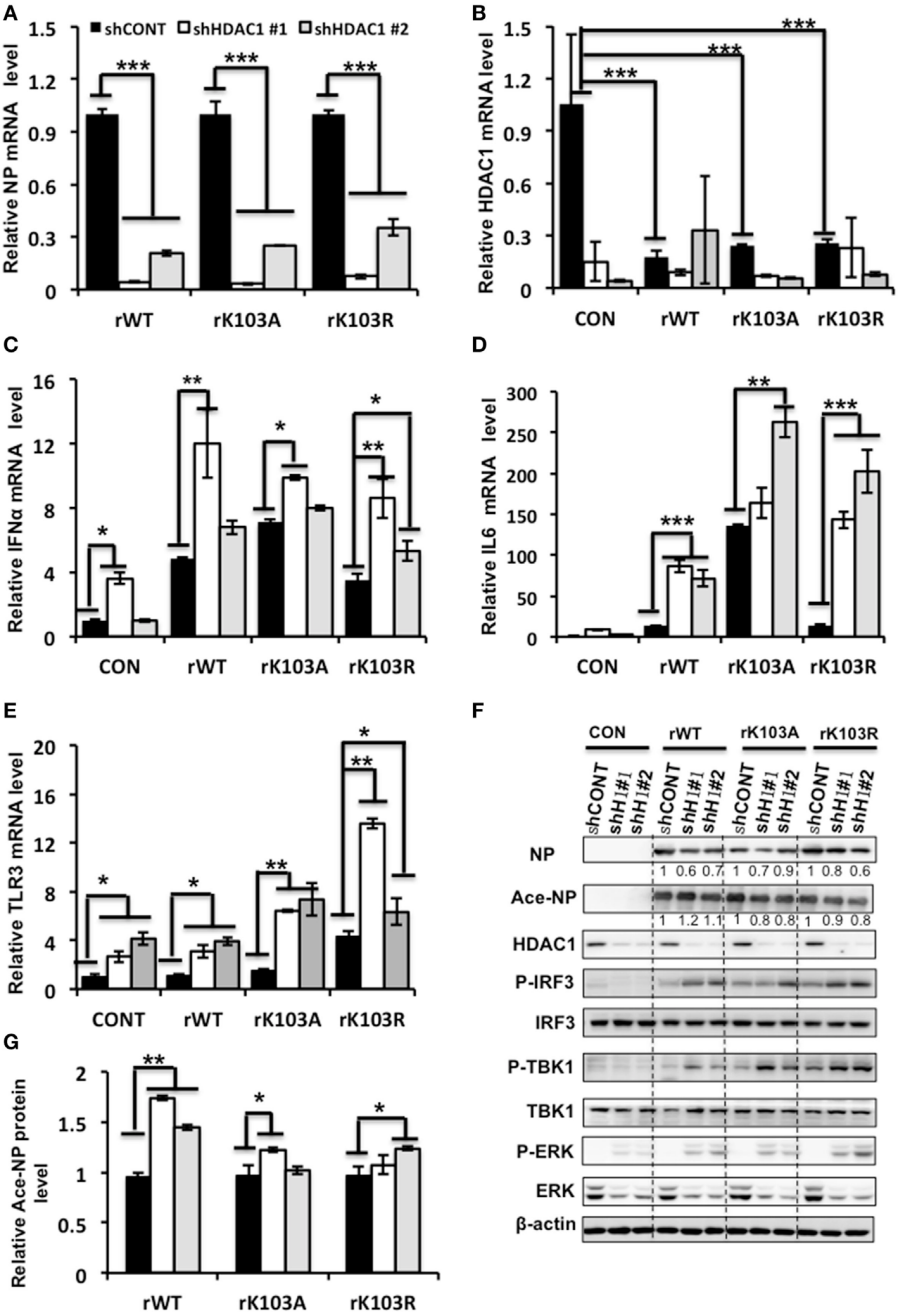
Depletion of histone deacetylase 1 (HDAC1) inhibited influenza A viruses (IAV) infection through activation the TBK1-IRF3 and ERK signaling pathways. A549 knockdown HDAC1 stable cells and control cells (shCONT) were infected with rWT/rK103A/rK103R virus at an MOI of 1 for 24 h. **(A–E)** The relative expression level of nucleoprotein (NP) **(A)**, HDAC1 **(B)**, IFNα **(C)**, IL6 **(D)**, and TLR3 **(E)** were determined by qRT-PCR. **(F)** Total lysate of the infected cells was subjected for Western blot analysis of viral NP, Ace-NP, HDAC1, p-IRF3, IRF3, p-TBK1, TBK1, p-ERK, and ERK. β-actin was used as a loading control. **(G)** The relative Ace-NP expression level equal to the Ace-NP level divided by the total NP. *P < 0.05; **P < 0.01, ***P < 0.001 (analysis of two-way ANOVA followed by Bonferroni post-test). Data are representative of three independent experiments [mean ± SD in **(A–E)**].

To further elucidate the molecular mechanism how HDAC1 regulates IAV virus production, we analyzed the gene expression profile of cells infected with rWT, rK103A, or rK103R influenza virus at an MOI of 1. The NP mRNA level was decreased in HDAC1-depleted cells compared to shCONT cells (Figure 6A). The HDAC1 mRNA level was downregulated when A549 control cells were infected with IAV (Figure 6B). The relative Ace-NP was increased in HDAC1-knockdown cells compared with the control cells (Figure 6G). It has been reported that type I IFN pathway that played a critical role in antivirus activity (37). Interestingly, gene expression analysis using qRT-PCR showed that IFNα mRNA levels were higher in HDAC1-depleted cells in response to the IAV infection (Figure 6C). The secretion of the IFNα in the culture supernatants was increased in HDAC1-depleted cells induced by IAV infection (Figure S2A in Supplementary Material). Results from siRNA-mediated knockdown of HDAC1 further confirmed these results (Figures S2B–E in Supplementary Material). To determine whether IRF3 has a role in producing IFN type I in the HDAC1-depleted cells after IAV infection, we measured the phosphorylation level of IRF3 in these cells. The results showed that phosphorylation of IRF3 were increased in HDAC1-depleted cells infected with IAVs compared with the control cells (Figure 6F). As TBK1 has been implicated in IRF3 activation in response to viral infections, we then test whether TBK1 is involved in IRF3 activation, thus leading to type I IFN production following infection with IAV. As shown in Figure 6F, the phosphorylation of TBK1 was increased in HDAC1-depleted cells infected with IAV compared with the control cells. Collectively, these results suggest that knockdown of HDAC1 suppresses IAV replication through activation of TBK1-IRF3 signaling pathway.

In addition, the TLR3 and IL6 mRNA levels were increased in HDAC1-depleted cells infection with IAV (Figures 6D,E). However, the mRNA levels of P53, TNF-α, and ILF16 mRNA levels did not differ significantly between the HDAC1-depleted and the control cells (Figure S3 in Supplementary Material). Moreover, the phosphorylation of ERK was increased in HDAC1-depleted cells infected with IAVs compared with the control cells (Figure 6F). On the contrary, the phosphorylation of AKT was decreased in HDAC1-depleted cells (Figure S4 in Supplementary Material). Collectively, knockdown of HDAC1 suppressed IAV replication through activation of the TBK1-IRF3 and ERK signaling pathways.

### NP-HDAC1 as a Switch for Activation Type I IFN Signaling Pathway

**Figure 7:**
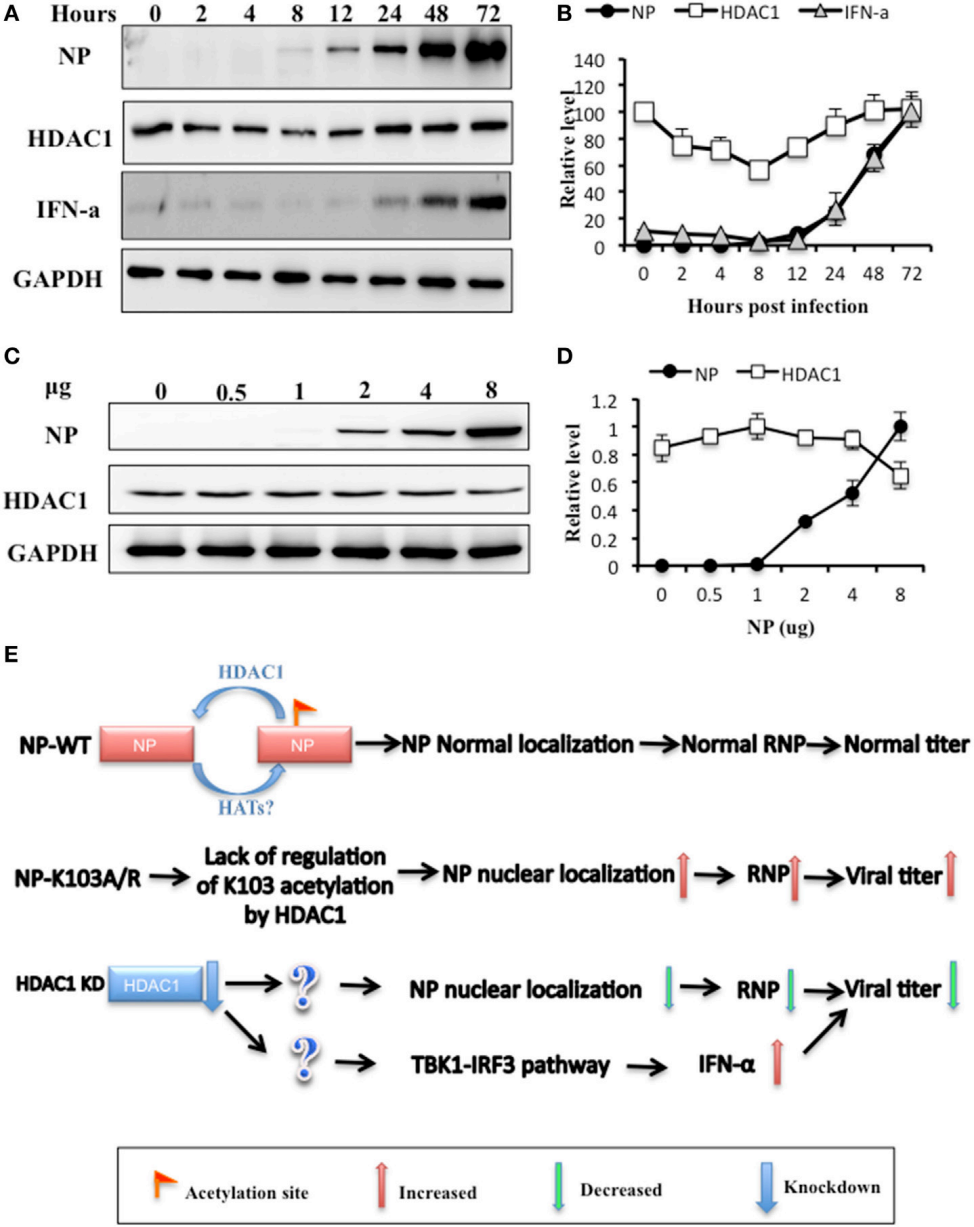
Histone deacetylase 1 (HDAC1) was a possible switch for regulation of the TBK1-IRF3 signaling pathways. **(A)** A549 cells were infected with H5N1 virus at an MOI of 0.5 and collected at indicated time postinfection. **(B)** Nucleoprotein (NP), HDAC1, and IFN-α levels were quantified relative to GAPDH and by densitometry using ImageJ. **(C,D)** HEK293T cells were transfected with gradient amount of NP for 36 h, protein **(C)**, and mRNA **(D)** level of HDAC1 and NP were analyzed. Data are representative of three independent experiments [mean ± SD in **(B,D)**]. **(E)** A possible schematic model: K103 of NP was an acetylation site regulated by HDAC1. Two K103 NP mutants, K103A and K103R, lacked of regulation by HDAC1, enhanced NP nuclear localization and replication efficiency of the recombinant viruses *in vitro*. Depletion of HDAC1 inhibited influenza A virus replication *via* Two paths: one was decreased the RNP activity, one was activated TBK1-IRF3 signaling.

To further determine the dynamic change of HDAC1 level after IAV infection, we analyzed the time course of HDAC1 expression profile in H5N1 infected cells at an MOI of 0.5. The cell lysates were harvested on 2, 4, 8, 12, 24, 48, and 72 h postinfection and detected HDAC1 and NP expression by Western blot. We found that the level of HDAC1 polypeptide was reduced at the early stage of IAV infection and reached a valley at 8 h (Figures 7A,B). However, the HDAC1 expression level gradually came back up to the basal level from 12 to 48 h and reached a plateau at 48 h (Figures 7A,B). Meanwhile, a time-dependent increase in the NP level was also observed. The host cells produced IFN-a at 8 h postinfection, and the level was increased in a time-depend manner (Figures 7A,B). We speculated that downregulation of HDAC1 at the early stage in IAV-infected cells might be a self-host protective response to the virus infection, which may be critical to activate the IFN-α expression.

To further determine the interaction between HDAC1 and NP, we transfected HEK293T cells with different concentration of NP. NP exhibited a dose-dependent effect on the HDAC1 level, which was downregulated with the increasing NP expression (Figures 7C,D). Based on our data and others, we propose a possible schematic model: in the NP WT, K103 of NP was an acetylation site regulated by HDAC1. Two K103 NP mutants, K103A and K103R, lacking of regulation of K103 acetylation by HDAC1, enhanced the RNPs activity and replication efficiency of the recombinant viruses *in vitro*. In addition, depletion of HDAC1 inhibited influenza A virus replication *via* two paths: one was decreased the RNP activity, and the other was activated TBK1-IRF3 signaling, which was independent the NP K103 site (Figure 7E).

## Discussion

Histone deacetylase 1 plays a crucial role in the transcription control of eukaryotic cells (38, 39). Intriguingly, the role of HDAC1 varied in different pathways, so much as in closely related pathways. For example, HDAC1-deficient cells exhibit decreased IFN-stimulated gene expression, however, increased IFN-β mRNA, suggesting that HDAC1 has unique and opposing roles in the regulation of the two related pathways of innate antiviral immunity (40). It seemed that the regulation of HDAC1 on the replication of virus followed a similar pattern in our study. We found that for one thing, the deacetylation of NP by HDAC1 at K103 increased the RNP activity and further promoted the IAV replication. For another, both the WT and the K103A/R mutants containing IAV showed same replication inhibition in HDAC1-deficient cells, indicating a K103 deacetylation-independent regulation of HDAC1 in viral replication.

We proposed that the depletion of HDAC1 disrupted the nucleus and cytoplasm distribution of NP independent of the K103 acetylation and further led to reducing RNPs activity. In addition, HDAC1 is widely involved in multiple pathways, its deficient may effect other signaling. For example, HDAC1 is an essential component in multisubunit protein complexes, which include NuRD, Sin3, NoDE, and COREST complexes (41–43). Depletion of HDAC1 might impair these complexes, and thus, effects the viral replication. HDAC1 was also reported to inhibit IAV entry by interfering with the microtubule-mediated endosomal transport (28). Depletion of HDAC1 also triggered the compensation mechanism that other HDACs, HDAC2, and HDAC6 etc. would be activated (23, 39). HDAC6 also plays an important role in viral replication. It has been shown that HDAC6 inhibits IAV release by downregulating the trafficking of viral components to the plasma membrane through acetylated microtubules (29). Others reported that HDAC6 plays a pivotal role in the release of viral capsids from the cytosolic surface of endosomes, the dissociation of M1 from vRNPs, and the dispersion of capsid components in the cytosol (44). Taking together, it is possible that viral replication was modulated by multiple pathways; therefore, its actual performance in response to HDAC1 absence was a compromise among all the participants. In this scenario, the K103 mutation, which eliminated HDAC1 mediated deacetylation, minimized the noise from other pathways, and better reflected the true performance of viral replication, the as long as the mutation.

Nucleoprotein, on the other hand, undergoes posttranslational modifications beside acetylation, including phosphorylation, ubiquitination, sumoylation, and ISGylation (13, 15–17). We also found that the Ace-NP was also affected by ubiquitination and sumoylation (Figure S5 in Supplementary Material). Ubiqutination site NP-K184R mutant and sumolylation substitution NP-K7R had a lower level of acetylation compared with that of NP-WT (Figure S5 in Supplementary Material). These results indicated that other modifications were also involved in NP acetylation regulation; however, the full function of these modifications and how they affect each other still needed to be researched.

Besides K103, we found five other lysines 91, 198, 227, 229, and 470 of NP could either be modified by acetylation using mass spectrometry. In our previous study, we investigated their functions in influenza virus life cycle (36). Of these, the K91R and K198R showed decreased the polymerase activity, and K91R and K198R mutants cannot yield recombinant viruses, while K227R viruses showed a similar virulence as rWT. Moreover, the replication efficiencies of K229R mutant viruses were attenuated *in vivo* and *in vitro*. Importantly, rK470R increased the virus’s virulence in both cell culture and animal model (36). We have also substituted all lysine (K) residues with arginine(R). However, six lysine-substituted virus was not viable in three independent experiments, suggesting all acetylation sites mutations in NP are critical for the viral life cycle (data not shown). All these results demonstrated that NP mutation played a crucial role in IAV virulence and pathogenicity. Our finding may facilitate the understanding of the biology of the IAVs, which may be helpful for the prediction and surveillance of IAV.

In summary, we demonstrated that K103 is an NP acetylation site, and a lysine to alanine or arginine mutation at the K103 site affected IAV virulence *in vitro*. In addition, NP interacted with HDAC1 *in vivo* and *in vitro*, and depletion of HDAC1 suppressed replication of IAV through two paths: one was impaired the nuclear retention of NP, and the other was activated TBK1-IRF3 pathway. These findings offer a new mechanism for regulating NP acetylation and possible approaches for downregulating HDAC1 levels, which together could provide potential strategies for controlling influenza virus.

## Author Contributions

LC, CW, and HH designed this project; LC performed the main experiments; LC, CW, and JL wrote and revised the manuscript; WS, WL, YH, and HA conducted part of the experiments; and WS, ML, NZ, and HD propagated and quantified the virus strain and gave helpful advice regarding the project.

## Conflict of Interest Statement

The authors declare that the research was conducted in the absence of any commercial or financial relationships that could be construed as a potential conflict of interest. The reviewer KS and handling editor MS declared their shared affiliation, and the handling editor states that the process nevertheless met the standards of a fair and objective review.
