# Supplementary material for: Histone Deacetylase 1 Plays an Acetylation-Independent Role in Influenza A Virus Replication
Source: Front Immunol. 2017 Dec 12;8:1757. doi: 10.3389/fimmu.2017.01757 (PMC5733105; doi:10.3389/fimmu.2017.01757)
Supplement: Supplementary file 1 [file Data_Sheet_1.PDF]

# *Supplementary Material*

# Histone Deacetylase 1 Plays an Acetylation Independent Role in Influenza A Virus Replication

Lin Chen[1,3#], Chengmin Wang[1#], Jing Luo[1#] , Wen Su[1,3], Meng Li[1,3], Na Zhao[1,3], Wenting Lv[1,3], Hamidreza Attaran[1] , Yapeng He[1], Hua Ding[2], Hongxuan He[1,*]

*Correspondence: hehx@ioz.ac.cn

## Supplementary Material and Methods

### Computer Modeling

The three-dimensional crystal structure of the NP (Protein Data Bank accession No. 2Q06) was used to illustrate the location of K103. This modeling was performed with the PyMOL, a molecular visualization system.

## Supplementary Figures and Tables

Figure S1, related to Figure 2

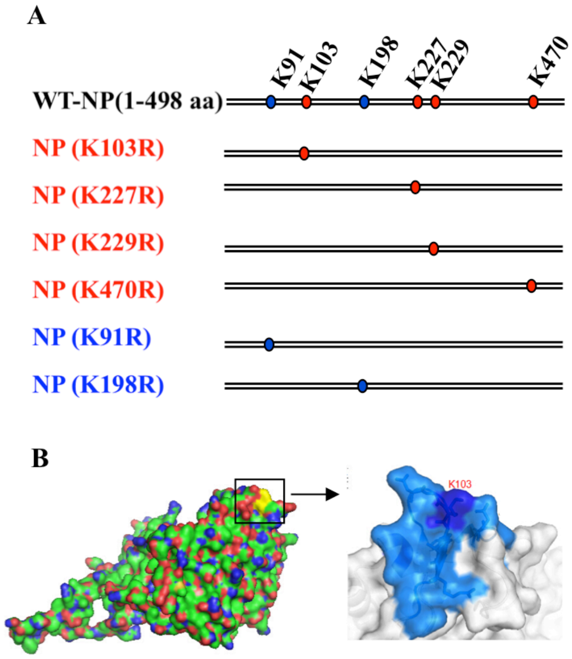


**Figure S2. NP Is Acetylated on the Lysine Residue at Amino Acid Position 103**. **(A)** Schematic representation of the NP with the positions of the lysines substituted with arginine residues in the different NP mutants. **(B)** The structure of the RBD showing the amino acids K103 was located on the surface of the NP. The RBD structure was generated from Protein Data Bank (identification no. 2Q06)

Figure S2

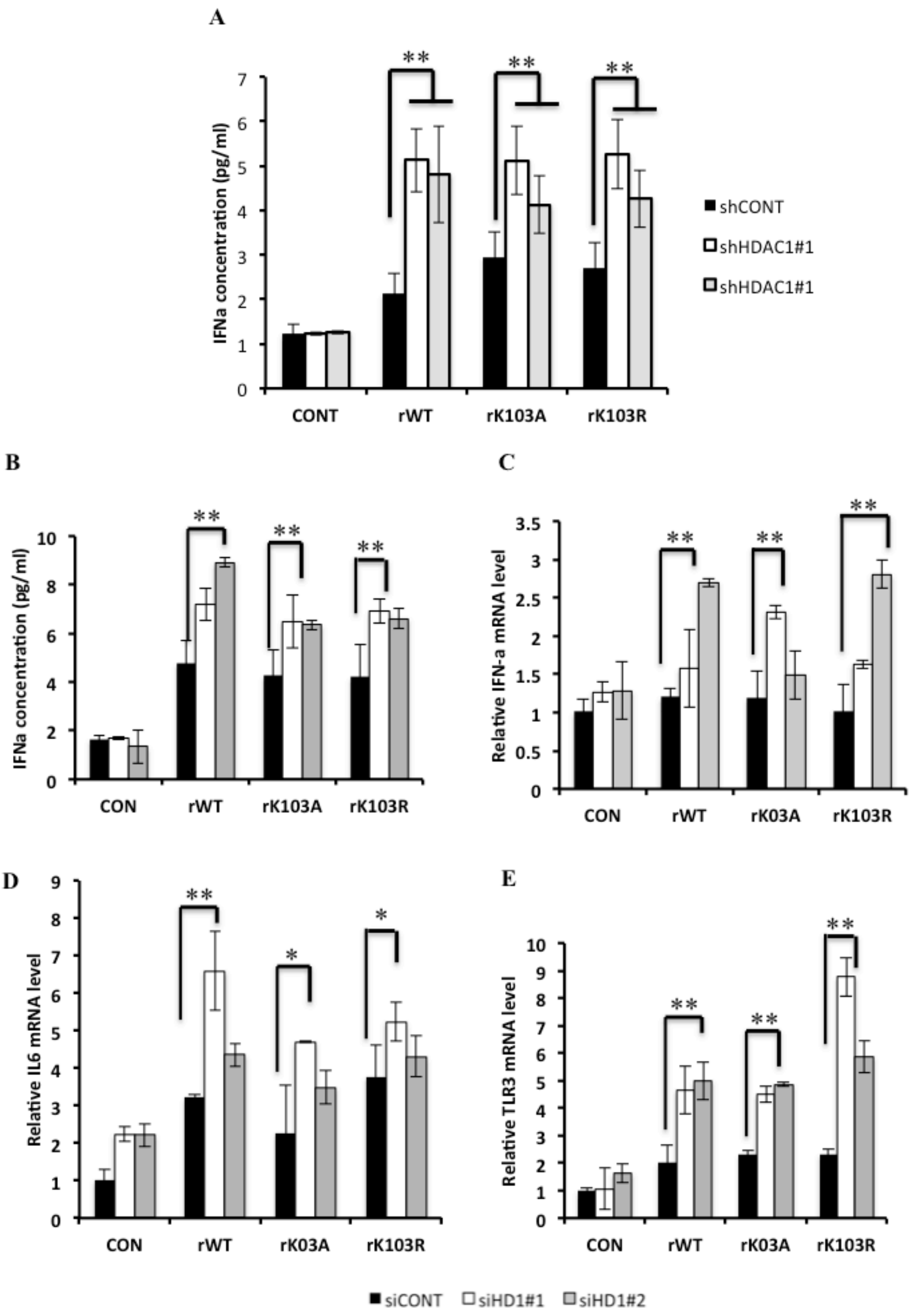


**FIGURE S2. Depletion of HDAC1 Inhibited IAV Infection through Activation the TBK1-IRF3 and ERK Signaling Pathways.**

**(A)** A549 Knockdown HDAC1 stable cells and shCONT cells were infected with rWT/rK103A/rK103R influenza virus at an MOI of 1 for 24 h. The amounts of IFNα released into the culture supernatant were measured by ELISA. **(B)** A549 cells were transfected with a non-targeting siRNA (siCONT), siHD1#1 or siHD1#2 for 72 h.

The cells were then infected with rWT, rK103A or rK103R virus at an MOI of 1 for 24 h. The amounts of IFNα released into the culture supernatant were measured by ELISA. **(C-E)** A549 cells were transfected with a non-targeting siRNA (siCONT), siHD1#1 or siHD1#2 for 72 h. The cells were then infected with rWT, rK103A or rK103R virus at an MOI of 1 for 24 h. Total RNA was isolated by using TRIzol Reagent. The amount of IFNα mRNA (C), IL6 mRNA (D) and TLR3 mRNA (E) were determined by qRT-PCR. The reactions were carried out in triplicate and normalized to the levels of β-actin. The value for shCONT cells was set to 1. Data represent mean ± SD values. *, $P < 0.05$; **, $P < 0.01$; ***, $P < 0.001$.

Figure S4, related to figure 6

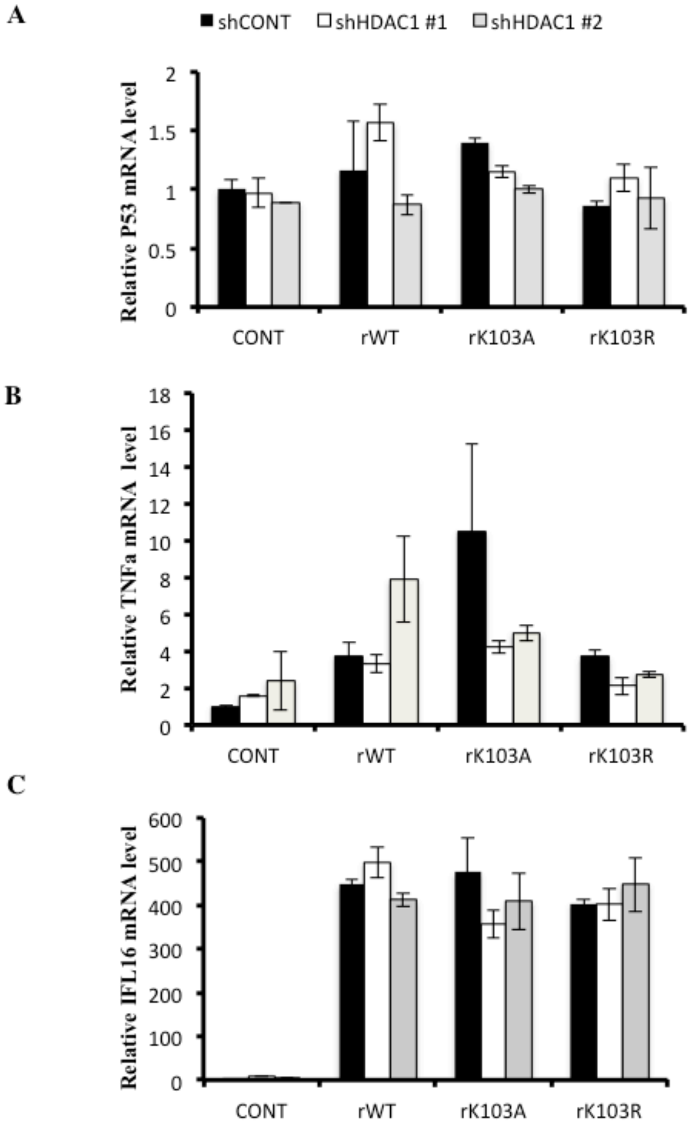


**Figure S3. The mRNA Expression Levels were Changed in HDAC1 Deficient**

**Cells Response to IAVs.** A549 Knockdown HDAC1 stable-cells and control cells (shCONT) were infected with rWT, rK103A, or rK103R virus at an MOI of 1 for 24 h. Total RNA was isolated by using TRIzol Reagent. The amount of the P53 mRNA (**A**), the TNF-α mRNA (**B**), and the IFL-16 mRNA (**C**) was determined by qRT-PCR. The reactions were carried out in triplicate and normalized to the levels of β-actin. The value for shCONT cells was set to 1. Data were shown as means ± s.d. The bars indicate s.d. of three independent experiments.

Figure S4.

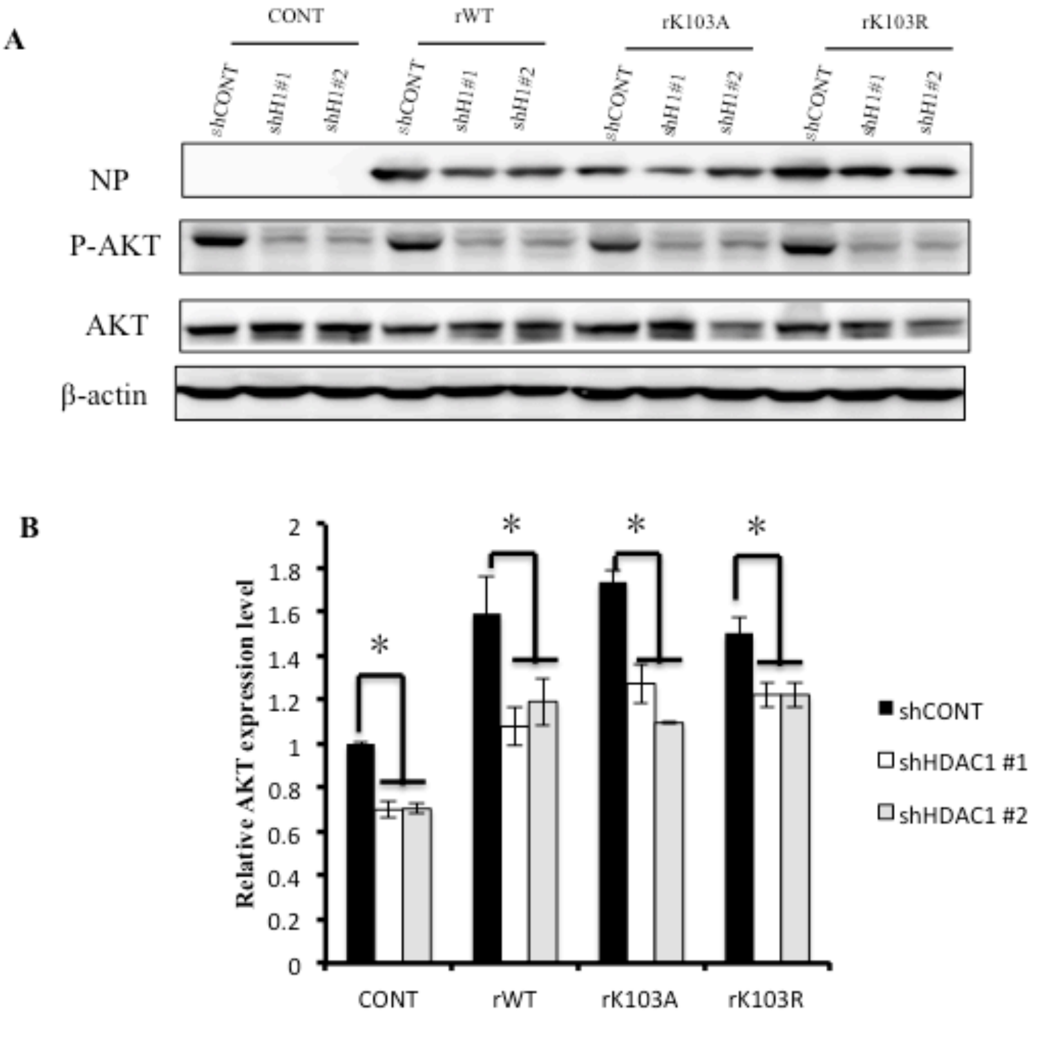


**Figure S4. The Phosphorylation Level of AKT was Inhibited in HDAC1-depletion Cells Infected with IAVs. (A)** A549 Knockdown HDAC1 stable cells and shCONT cells were infected with rWT/rK103A/rK103R influenza virus at an MOI of 1 for 24 h. Total lysate of the infected cells was prepared, and viral NP, p-AKT and AKT were detected by Western blot. β-actin was used as a loading control.

CONT, control.

**(B)** A549 Knockdown HDAC1 stable cells and control cells (shCONT) were infected with rWT/rK103A/rK103R virus at an MOI of 1 for 24 h. Total RNA was isolated by using TRIzol Reagent. The AKT mRNA level was determined by qRT-PCR. The reactions were carried out in triplicate and normalized to the levels of β-actin. The value for shCONT cells was set to 1. Data represent mean ± SD values.

Figure S5.

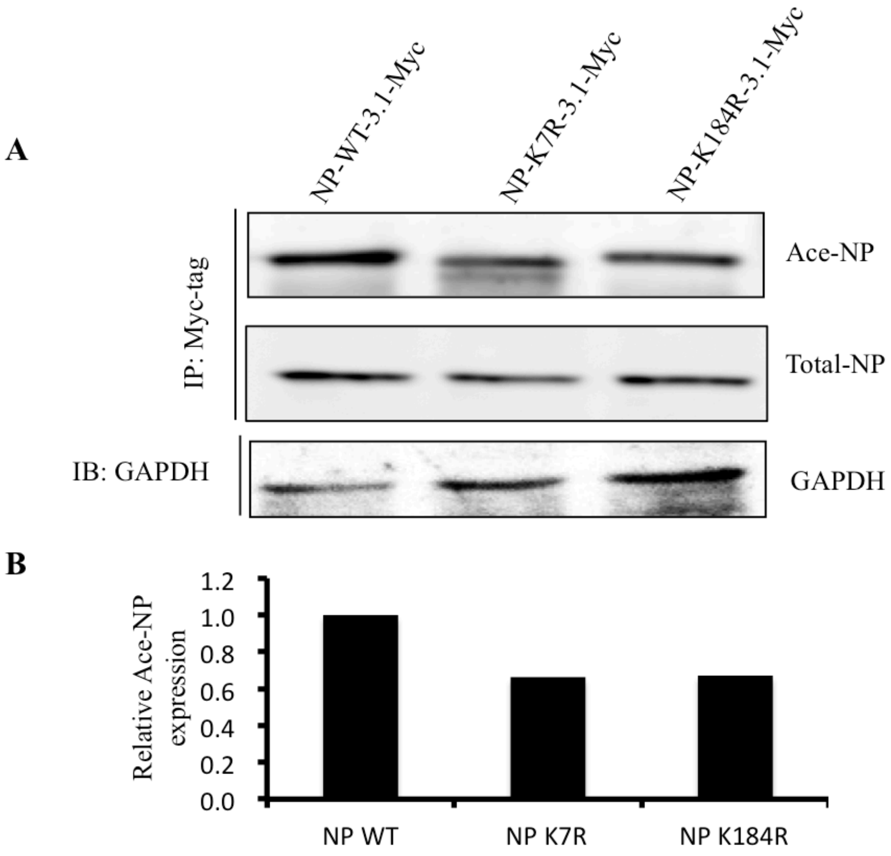


**Figure S5. Acetylation Level of NP was Affected by Ubiquitination and Sumoylation.**

(**A**) HEK293T cells were transfected with vectors expressing WT or mutant NP-Myc. The cell extracts were purified by Myc-tagged pull-down followed by Western blotting (IB) of the anti-acetylated-lysine (Ace-Lys) antibodies; GAPDH was used as a loading control. IP, immunoprecipitation.

(**B**) The relative Ace-NP protein expression level was quantified by using imageJ.

**Table S1. Primers used in this study**

| Primer name | Sequence（5′to 3′） |
|---|---|
| NP-CDS-F | CCCGGATCCATGGCGTCTCAGGGCACCAAACGA |
| NP-CDS-R | CCCCTCGAGATTGTCATATTCCTCTGCATTGTC |
| NP-K91R-F | GGACCCAAAGAGAACTGGAGGTCCAATCTACC |
| NP-K91R-R | GGTAGATTGGACCTCCAGTTCTCTTTGGGTCC |
| NP-K103R-F | AGAGACGGGAGGTGGATAAGAGAGCTGATTCT |
| NP-K103R-R | AGAATCAGCTCTCTTATCCACCTCCCGTCTCT |
| NP-K198R-F | TCGGATGATAAGACGAGGGATCAATGATCG |
| NP-K198R-R | CGATCATTGATCCCTCGTCTTATCATCCGA |
| NP-K227R-F | CAACATCCTCAGAGGGAAATTCCAAACAGCAG |
| NP-K227R-R | CTGCTGTTTGGAATTTCCCTCTGAGGATGTTG |
| NP-K229R-F | CCTCAAAGGGAGATTCCAAACAGCAGCACA |
| NP-K229R-R | TGTGCTGCTGTTTGGAATCTCCCTTTGAGG |
| NP-K470R-F | CTCTCGGACGAAAGGGCAACGAACCCGATC |
| NP-K470R-R | GATCGGGTTCGTTGCCCTTTCGTCCGAGAG |
| NP-K103A-F | AGAGACGGGGCCTGGATAAGAGAGCTGATTCT |
| NP-K103A-R | AGAATCAGCTCTCTTATCCAGGCCCCGTCTCT |
| HDAC1-CDS-F | CCCGAATTCATGGCGCAGACGCAGGGCACCCGG |
| HDAC1-CDS-R | CCCCTCGAGTCAGGCCAACTTGACCTCCTC |
| HDAC2-CDS-F | CGCGGATCCATGGCGTACAGTCAAGGAGGC |
| HDAC2-CDS-R | CCGCTCGAGTCAGGGGTTGCTGAGCTGTTC |
| HDAC3-CDS-F | CCCGAATTCATGGCCAAGACCGTGGCCTAT |
| HDAC3-CDS-R | CCCTCTAGATTAAATCTCCACATCGCTTTC |
| HDAC8-CDS-F | CCCGAATTCATGGAGGAGCCGGAGGAACCG |
| HDAC8-CDS-R | CCCCTCGAGCTAGACCACATGCTTCAGATT |
| IFNα-F | CTTGACTTGCAGCTGAGCAC |
| IFNα-R | CAGAGTCACCCATCTCAGCA |
| IL6-F | GTCAGGGGTGGTTATTGCAT |
| IL6-R | AGTGAGGAACAAGCCAGAGC |
| TLR3-F | TTTGATGAGTCTCCGCCAACTCCA |
| TLR3-R | AATCTGCGAGGGACACAAAGGTCT |
| AKT-F | TGAAGGTGCCATCATTCTTG |
| AKT-R | ATGAGCGACGTGGCTATTGT |
